# Blocking soluble guanylate cyclase could be the present and future of NO/cGMP inhibition for vasoplegia treatment

**DOI:** 10.1186/s13054-018-2024-y

**Published:** 2018-04-20

**Authors:** Paulo Roberto Barbosa Evora

**Affiliations:** Department of Surgery and Anatomy (Division of Cardiothoracic Surgery); Ribeirão Preto School of Medicine, University of São Paulo, Rua Rui Barbosa, 367, Ap 15, Rbeirão Preto, SP 14015-120 Brazil

Cumulative evidence indicates that NO/cGMP inhibition would make a difference in vasoplegia treatment. Unfortunately, methylene blue (MB) is the only compound we currently have that can be used for this purpose. The effects of MB are apparent only in the case of nitric oxide (NO) up-regulation, and it is not a vasoconstrictor per se. Blocking the cGMP system “releases” the cAMP system in a kind of “crosstalk,” facilitating the noradrenaline vasoconstrictor effect [1, 2].

I recently wrote an editorial opinion suggesting this approach as an unexplored therapeutic frontier for the pharmaceutical industry. MB is the oldest drug infused in humans, is cheap, and is not patented for industrial use, but the medical literature insists that it has not been the subject of any sizeable clinical trials, even considering its safety at low doses. However, it is mandatory to use it early, not as rescue therapy [3].

Studies suggest that MB has a potential role in protecting the microcirculation. In an experimentally induced septic shock model in rats, only the combination of norepinephrine (NE) and MB restored mean arterial pressure to control levels by the end of the 3-h experiment. Intravital microscopy demonstrated better microvascular integrity in the presence of MB, and severe damage to animals that were infused with only NE [4]. Therefore, MB would be an option to prevent direct organ damage from adrenergic agents.

Levy and colleagues wrote an excellent review article about the “past, present and future” of vasoplegia treatment and presented some concerns about MB use [5]. Not considering MB itself, blocking soluble guanylate cyclase (sGC), the final NO/cGMP messenger, in smooth muscle is underestimated and deserves a place in the present and future.

## Abbreviations

cAMP: Cyclic adenosine monophosphate
cGMP: Cyclic guanosine monophosphate
MB: Methylene blue
NO: Nitric oxide
